# Mucosal Challenge Ferret Models of Ebola Virus Disease

**DOI:** 10.3390/pathogens10030292

**Published:** 2021-03-04

**Authors:** Trevor Brasel, Jason E. Comer, Shane Massey, Jeanon Smith, Jennifer Smith, Matthew Hyde, Andrew Kocsis, Melicia Gainey, Nancy Niemuth, Cheryl Triplett, Thomas Rudge

**Affiliations:** 1 Department of Microbiology and Immunology, University of Texas Medical Branch, 301 University Blvd., Galveston, TX 77573, USA; jscomer@utmb.edu (J.E.C.); chmassey@utmb.edu (S.M.); jensmit1@utmb.edu (J.S.); 2 Department of Pathology, University of Texas Medical Branch, 301 University Blvd., Galveston, TX 77573, USA; jeksmith@utmb.edu; 3 Animal Resources Center, University of Texas Medical Branch, 301 University Blvd., Galveston, TX 77573, USA; mahyde@utmb.edu (M.H.); agkocsis@utmb.edu (A.K.); 4 Battelle, 1425 Plain City-Georgesville Road, NE, West Jefferson, OH 43162, USA; GaineyM@battelle.org (M.G.); niemuth@battelle.org (N.N.); triplettc@battelle.org (C.T.); rudget@battelle.org (T.R.J.)

**Keywords:** Ebola virus, ferret, mucosal challenge

## Abstract

Recent studies have shown the domestic ferret (*Mustela putorius furo*) to be a promising small animal model for the study of Ebola virus (EBOV) disease and medical countermeasure evaluation. To date, most studies have focused on traditional challenge routes, predominantly intramuscular and intranasal administration. Here, we present results from a non-clinical pathogenicity study examining oronasal, oral, and ocular mucosal challenge routes in ferrets. Animals were challenged with 1, 10, or 100 plaque forming units EBOV followed by monitoring of disease progression and biosampling. Ferrets administered virus via oronasal and oral routes met euthanasia criteria due to advanced disease 5–10 days post-challenge. Conversely, all ferrets dosed via the ocular route survived until the scheduled study termination 28-day post-challenge. In animals that succumbed to disease, a dose/route response was not observed; increases in disease severity, febrile responses, serum and tissue viral load, alterations in clinical pathology, and gross/histopathology findings were similar between subjects. Disease progression in ferrets challenged via ocular administration was unremarkable throughout the study period. Results from this study further support the ferret as a model for EBOV disease following oral and nasal mucosa exposure.

## 1. Introduction

Ebola virus (EBOV) is a single-stranded, negative-sense RNA virus of the order *Mononegavirales* that can cause an acute, often fatal, disease in humans. EBOV first appeared in 1976 during an outbreak that claimed almost 300 lives in the Democratic Republic of the Congo (DRC). Since then, it has been reported in numerous outbreaks including the 2014–2015 West African outbreak and the 2018–2020 outbreak in the DRC which resulted in over 11,000 and close to 2300 deaths, respectively. In December 2019, Ervebo became the first U.S. Food and Drug Administration- and European Medicines Agency-approved vaccine for the prevention of EBOV disease (https://www.fda.gov/vaccines-blood-biologics/ervebo accessed on 20 December 2019; https://www.ema.europa.eu/en/medicines/human/EPAR/ervebo accessed on 20 December 2019). In July 2020, Mvabea and Zabdeno were approved for similar indications (https://www.ema.europa.eu/en/medicines/human/EPAR/mvabea accessed on 1 July 2020; https://www.ema.europa.eu/en/medicines/human/EPAR/zabdeno accessed on 1 July 2020). Although effective vaccines now exist, there is still a great need to develop and test additional countermeasures to minimize disease in future, imminent outbreaks.

Animal models have been critical in the development and testing of medical countermeasures (MCM) for EBOV. Studies have clearly shown that nonhuman primates (NHPs) are ideal for studying EBOV pathogenesis and MCMs since they uniformly develop clinical disease and signs characteristic of EBOV disease observed in humans [1,2,3,4,5]. Given the complexity associated with NHP work, particularly in a Biosafety Level 4 environment, and the current and future shortage of NHP supply [6], alternative models are highly desirable. While small animal models are useful tools, studies have shown that MCM efficacy does not necessarily translate to efficacy in higher order animals, including NHPs [7]. In addition, numerous small animal models have been hindered because adapted viruses (e.g., mouse- or guinea pig-adapted variants) must be used and, importantly, the resulting diseases do not recapitulate disease in NHPs or humans [8].

Recent studies have shown the domestic ferret (*Mustela putorius furo*) to be a promising animal model for the study of EBOV disease and MCM evaluation. Ferrets are susceptible to wild-type (i.e., non-adapted) EBOV and demonstrate clinical disease similar to NHPs and humans [7,9,10,11,12,13]. Further, ferrets have been used to evaluate the protective efficacy of novel MCMs [14] and thus are a promising model in lieu of NHPs. To date, most studies performed in ferrets have focused on traditional challenge routes, predominantly intramuscular and intranasal administration [9,15]. In humans, EBOV spreads via direct contact through broken skin or mucous membranes in the eyes, nose, and/or mouth. While intranasal challenge in ferrets recapitulates EBOV disease following nasal mucosa exposure in humans, other mucosal routes warrant investigation. To address this need, we conducted a non-clinical pathogenicity study examining ocular and oral mucosal routes of infection.

## 2. Results

### 2.1. Virus Challenge and Post-Challenge Disease Presentation

**Figure 1:**
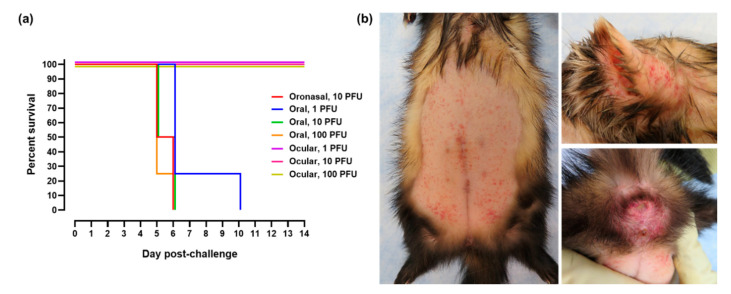
Survival and petechial rash following EBOV mucosal challenge. (**a**) Kaplan–Meier survival curve following oronasal, oral, and ocular virus challenge. For graph clarity and separation of the acute disease phase, Days 15–28 have been removed. (**b**) Representative images of petechial rash noted in ferrets administered EBOV via oronasal and oral routes. Rash was prominent on the abdomen (left image), head/ears (top right), and perianal surface (bottom right).

Three mucosal routes of EBOV challenge were investigated in this study (oronasal, oral, and ocular). Ferrets randomized into the oronasal challenge group served as controls for disease progression based on work first presented in 2015 [16] and were administered a target dose of 10 plaque forming units (PFU). Animals placed into the oral and ocular challenge groups were administered target doses of 1, 10, or 100 PFU. Based on plaque assay data, the mean challenge dose for the oronasal group was 13.3 PFU. For ferrets challenged via the oral route, the mean challenge doses were 1.2, 7.8, and 76.6 PFU for the 1, 10, and 100 PFU target dose groups, respectively. For ferrets challenged via the ocular route, the mean challenge doses were 0.8, 9.9, and 90.0 PFU for the 1, 10, and 100 PFU target groups, respectively. Ferrets challenged via oronasal and oral administration required euthanasia 5–10 days post-challenge (Figure 1a). The median times to death following oronasal challenge with 10 PFU and oral challenge with 1, 10, and 100 PFU were 136, 152, 136, and 126 h, respectively. Survival times were not statistically different between these groups at a 0.05 level of significance. All animals administered virus via the ocular route survived until scheduled study termination on Day 28. Petechial rash characteristic of EBOV disease (Figure 1b) was observed in all ferrets challenged via oronasal and oral routes. Severity of the rash was equivalent in all animals. Petechial rash was absent in all ferrets following ocular challenge. Additional clinical signs commonly observed in the ferrets administered EBOV via oronasal and oral routes included general lethargy and inactivity, unkempt appearance, increased respiration (>50 breaths per minute), and diarrhea/loose stool (Table 1). Less common signs included visible ecchymosis and respiration rate greater than 80 breaths per minute (data not shown). Outward clinical disease was absent in ferrets challenged via the ocular route.

**Figure 2:**
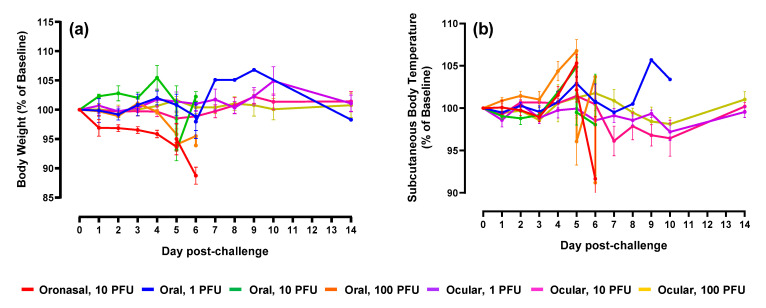
Body weight and subcutaneous temperature alterations following EBOV mucosal challenge. Percent change in body weight (**a**) and subcutaneous body temperature (**b**) from Day 0. For graph clarity and separation of the acute disease phase, Days 15–28 have been removed; alterations were minimal for remaining animals during this period of time. For both graphs, error bars represent standard error of the mean.

**Figure 3:**
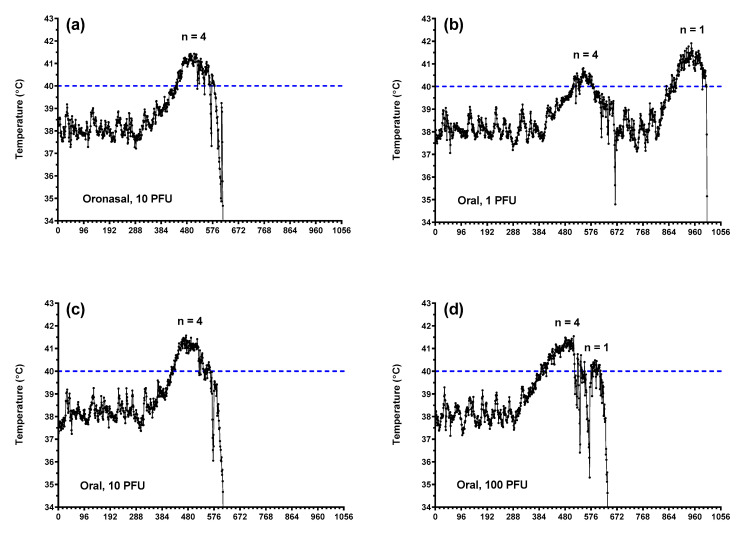
Core body temperature alterations in ferrets challenged with EBOV via oronasal and oral routes. Core body temperatures as measured via implanted Star–Oddi DST micro-T implantable temperature loggers following oronasal (**a**) and oral (**b**–**d**; 1, 10, and 100 PFU, respectively) challenge. Each tick on the *x*-axis represents 4 h or 16 individual logger measurements.

**Figure 4:**
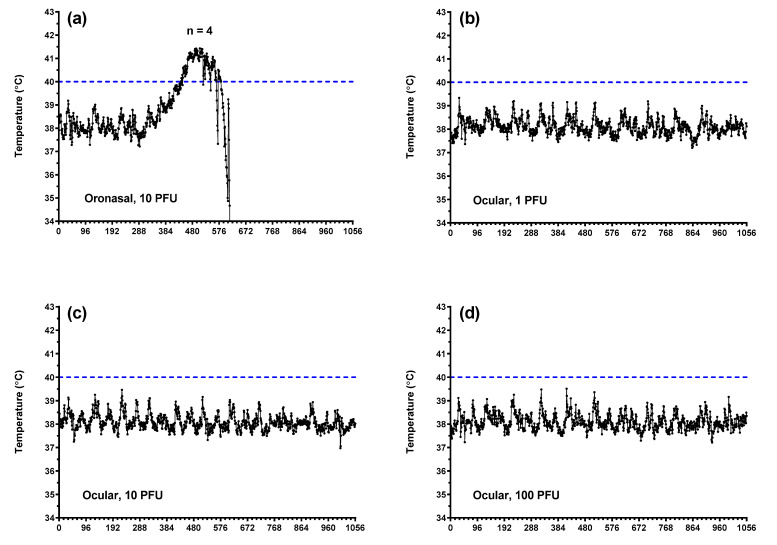
Core body temperature alterations in ferrets challenged with EBOV via oronasal and ocular routes. Core body temperatures as measured via implanted Star–Oddi DST micro-T implantable temperature loggers following oronasal (**a**) and ocular (**b**–**d**; 1, 10, and 100 PFU, respectively) challenge. Each tick on the *x*-axis represents 4 h or 16 individual logger measurements.

In general, body weight values decreased following oronasal and oral virus administration, although this was not observed consistently across groups (Figure 2a). Subcutaneous body temperatures (Figure 2b) aligned with core temperatures as measured using implanted Star–Oddi temperature loggers (Figure 3 and Figure 4). Fevers, defined as two consecutive hours (eight consecutive measurements) of temperatures greater than 40 °C, were measured consistently in ferrets following oronasal (Figure 3a and Figure 4a) and oral (Figure 3) virus challenge. In the low dose oral challenge group (Figure 3b), a delayed febrile response was observed in one animal. For these groups, the median time to onset of elevated temperature decreased as virus dose increased and was slightly longer, though not statistically significant, for the oronasal route compared to the oral route. Body weights and temperatures remained relatively unchanged in the ocular challenge groups.

### 2.2. Clinical Pathology and Serum Viral Load

**Figure 5:**
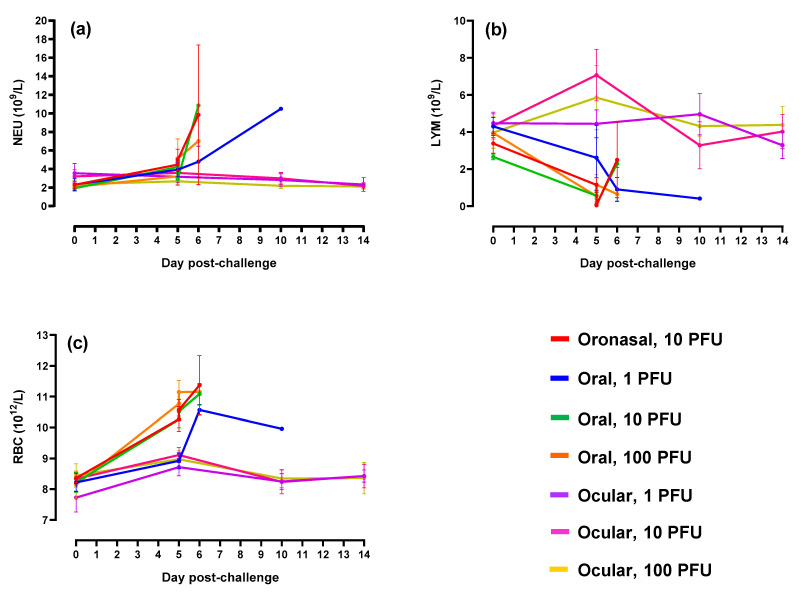
Hematology parameters. (**a**–**c**) Neutrophil (NEU), lymphocyte (LYM), and red blood cell counts (RBC), respectively. For graph clarity and separation of the acute disease phase, Days 15–28 have been removed; alterations were minimal for remaining animals during this period of time. For all graphs, error bars represent standard error of the mean.

To evaluate the impact of mucosal EBOV challenge on immune cell populations and normal blood function, including coagulation, standard hematology analyses were performed. In ferrets administered virus via oronasal and oral routes, increases in neutrophils and red blood cells were observed in parallel with decreased lymphocyte counts (Figure 5). Alterations in animals belonging to the low dose (i.e., 1 PFU) oral challenge group were delayed and/or less severe as compared to the other three groups. The increased neutrophil counts combined with a decrease in lymphocytes was likely due to acute inflammation and depletion of inflammatory cell pools, respectively. Elevations in red blood cells were likely the result of dehydration, a commonly observed outcome in ferrets in advanced stages of EBOV disease. Of interest were the increases in lymphocytes (an indication of successful virus infection) observed on Day 5 in animals challenged via the ocular route at 10 and 100 PFU. Alterations in these animals were otherwise minimal.

**Figure 6:**
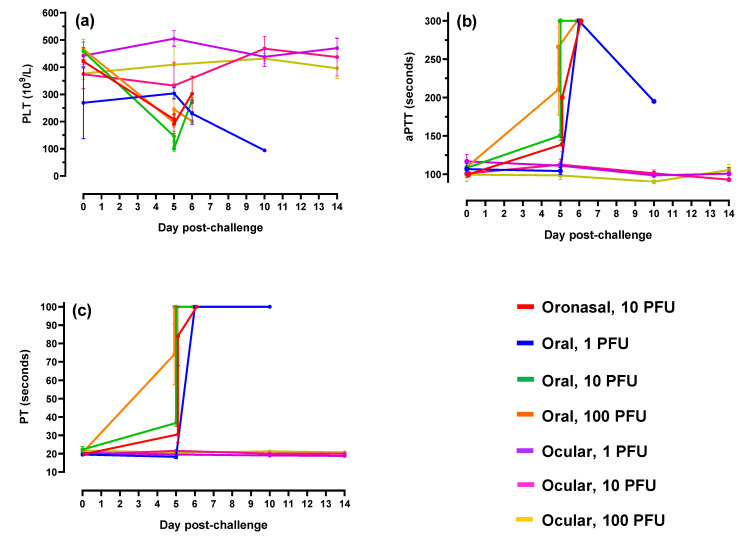
Coagulation parameters. (**a**–**c**) Platelet counts (PLT), activated partial thromboplastin time (aPTT) and prothrombin time (PT), respectively. For graph clarity and separation of the acute disease phase, Days 15–28 have been removed; alterations were minimal for remaining animals during this period of time. For all graphs, error bars represent standard error of the mean.

With respect to coagulation parameters, ferrets challenged via oronasal and oral routes demonstrated decreases in platelet counts in parallel with sharp increases in activated partial thromboplastin (aPTT) and prothrombin time (PT; Figure 6). These results were indicative of coagulation cascade disruption and advanced EBOV disease.

**Figure 7:**
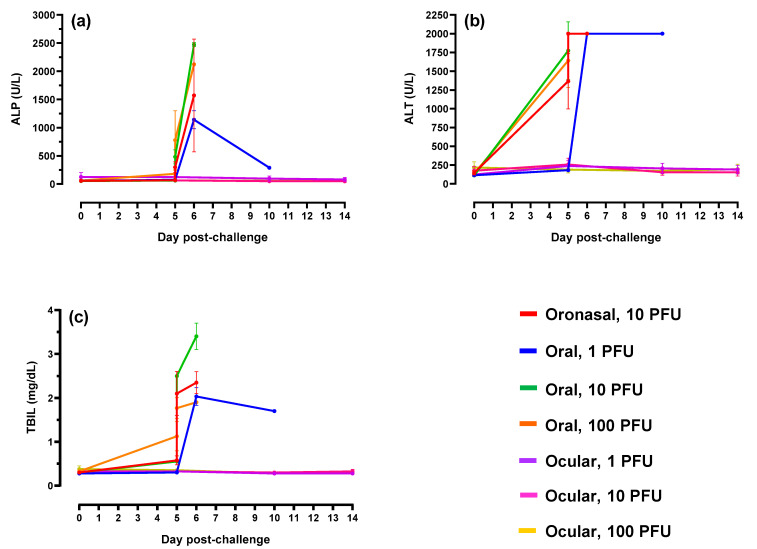
Liver biochemical profiles. (**a**–**c**) Alkaline phosphatase (ALP), alanine aminotransferase (ALT), and total bilirubin (TBIL), respectively. For graph clarity and separation of the acute disease phase, Days 15–28 have been removed alterations were minimal for remaining animals during this period of time. For all graphs, error bars represent standard error of the mean.

**Figure 8:**
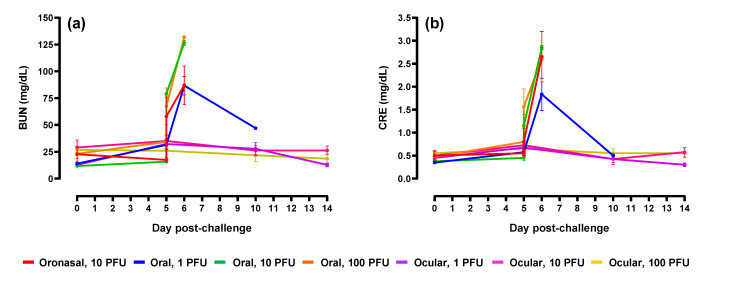
Kidney biochemical profiles. (**a**) Blood urea nitrogen (BUN) and (**b**) creatinine (CRE). For graph clarity and separation of the acute disease phase, Days 15–28 have been removed alterations were minimal for remaining animals during this period of time. For all graphs, error bars represent standard error of the mean.

Liver and kidney function is adversely impacted in humans and animal models of EBOV disease [4,17,18]. To assess the impact following mucosal challenge in ferrets, analysis of serum biochemistry was performed. Increases in the liver parameters alkaline phosphatase, alanine aminotransferase, and total bilirubin (Figure 7) and kidney parameters blood urea nitrogen and creatinine (Figure 8) were measured in animals following oronasal and oral virus administration; values were highest at peak disease (Day 5 post-challenge) which included terminal or near-terminal samples. Alterations were the result of acute liver inflammation, impaired kidney function, and/or dehydration. Compared to baseline, most of the noted increases were significant (*p* < 0.05). Alterations to these parameters were minimal in ferrets challenged via the ocular route.

**Figure 9:**
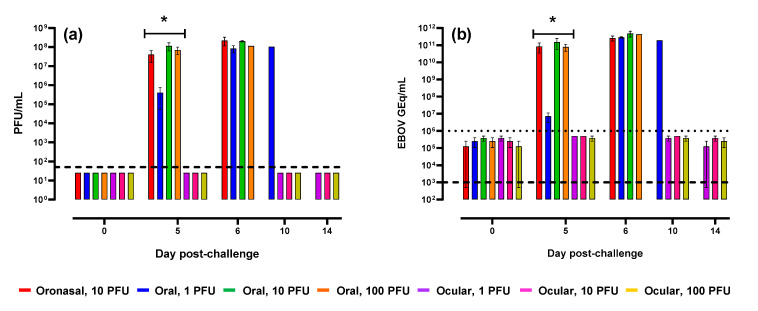
Serum viral load. EBOV levels in serum was determined via plaque assay (**a**) and qRT-PCR (**b**). Error bars represent standard error of the mean. Dashed lines represent the limit of detection (LOD) for each assay. For plaque assay, samples that lacked detectable plaques were assigned a value equivalent to one-half the LOD or 25 PFU/mL. The dotted line in panel (**b**) represents the lower limit of quantitation (LLOQ) for the qRT-PCR assay; a LLOQ was not defined for the plaque assay. For graph clarity and separation of the acute disease phase, Days 21 and 28 have been removed; values in remaining animals were below the LOD and/or LLOQ for the plaque and qRT-PCR assays, respectively. On Day 5, the mean increase as a proportion of baseline was significantly (*p* < 0.0001) greater in ferrets challenged via oronasal and oral routes (*) versus those challenged via ocular administration.

To assess viremia following mucosal challenge, serum was analyzed for infectious virus and viral RNA via plaque and qRT-PCR assays, respectively. Plaque assay results (Figure 9a) demonstrated high concentrations of virus at peak disease 5–10 days post-challenge in animals challenged via oronasal and oral routes. On Day 5, the mean increase as a proportion of baseline was significantly (*p* < 0.0001) greater in these groups as compared to ferrets challenged via the ocular route in which all tested samples lacked detectable virus. Results from qRT-PCR analysis (Figure 9b) were similar, although additional assay background attributed to non-specific amplification was noted in samples collected from ferrets challenged via the ocular route.

### 2.3. Tissue Viral Load, Gross Pathology, and Histopathology

**Figure 10:**
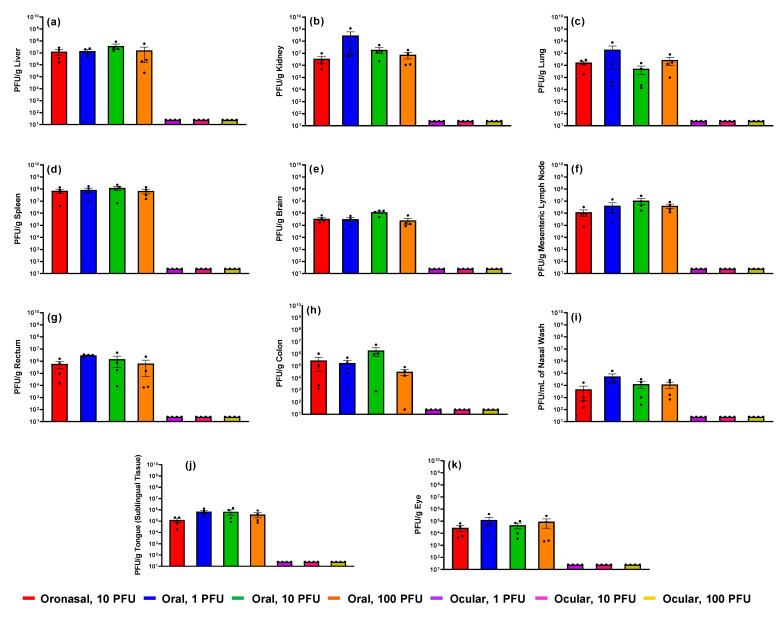
Infectious viral loads in tissue. From each ferret, select tissues and nasal wash were collected and analyzed for EBOV via plaque assay. (**a**) Liver; (**b**) kidney; (**c**) lung; (**d**) spleen; (**e**) brain; (**f**) mesenteric lymph node; (**g**) rectum; (**h**) colon; (**i**) nasal wash; (**j**) tongue [sublingual tissue]; (**k**) eye. Error bars represent standard error of the mean. Each dot (•) represents an individual animal in the group. PFU/g, EBOV plaque forming units per gram of tissue. Samples that lacked detectable plaques were assigned a value of 25 PFU/g (or mL for nasal wash).

Select tissues and nasal wash were collected from all ferrets at euthanasia (unscheduled and scheduled) for viral load analysis via plaque assay and qRT-PCR. Results (Figure 10 and Figure S1) demonstrated high concentrations of EBOV in the majority of samples collected from ferrets challenged via oronasal and oral routes with no significant differences between the two routes or challenge doses. Viral loads were highest in the liver, kidney, and spleen. In animals administered virus via the ocular route, all samples (collected at study termination 28 days post-challenge) lacked infectious virus, although quantifiable EBOV RNA was measured in the liver and lung of one animal (Figure S1).

**Figure 11:**
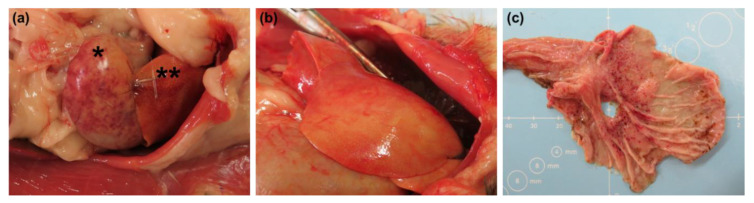
Diffuse, pinpoint, red discoloration was noted in the spleen ((**a**)**,** noted with an asterisk [*]) and stomach (**c**) of numerous ferrets following oronasal and oral EBOV challenge. The liver was noted as pale/yellow in all ferrets administered virus via these two mucosal routes ((**a**), noted with two asterisks [**] and (**b**)).

Gross and histopathological findings were predominantly associated with ferrets challenged via oronasal and oral routes (Table 2 and Table 3; Figure 11). These animals presented with pale, yellow livers (corresponding to inflammation and necrosis), enlarged lymph nodes, mottled dark red lungs (corresponding to alveolar/perivascular inflammation, edema, and/or necrosis), and diffuse, pinpoint, red discoloration in various tissues (e.g., skin, bladder mucosa, stomach mucosa, spleen, rectum, and thymus) associated with petechiation and/or hemorrhage commonly observed in animal models of EBOV disease. For all EBOV-related findings in these two challenge route groups, incidence and severity were similar between animals and virus doses. Findings in ferrets challenged via the ocular route were minimal or altogether absent, although liver-, lung-, and/or spleen-associated inflammation was noted upon histopathological examination in numerous animals in the 10 and 100 PFU challenge groups (Table 3).

## 3. Discussion

Despite the recent availability of approved vaccines, there continues to be a long-standing need for additional medical countermeasures to combat Ebola virus and similar filoviruses (e.g., Sudan, Bundibugyo, and Tai Forest viruses) for the prevention and treatment of disease in humans. As outbreaks are sporadic, advanced development and approval of new countermeasures will likely rely on well-characterized animal models. To date, numerous animal models, ranging from simple rodent screening models to pivotal nonhuman primate models, have been used to study Ebola virus disease and advance products including vaccines and therapeutics. Many of these models display clinical hallmarks of disease observed in humans, but often involve virus challenge routes that are not representative of natural infection (intramuscular versus mucosal, for instance). While nonhuman primates, in particularly macaques, appear to fully recapitulate key features of disease, they are in short supply and pose significant ethical challenges. To address these issues, we proceeded to develop and characterize ferret models of mucosal EBOV challenge.

Given the extensive characterization of intranasal EBOV challenge in the available literature [7,9,19], we focused our efforts on two alternative mucosal challenge routes, namely oral and ocular delivery. In an attempt to modify disease severity as seen in humans, three doses of virus (1, 10, and 100 PFU) were implemented. For infection control purposes, a small number of animals were challenged with 10 PFU via the oronasal route, a dosing regimen previously shown to be 100% lethal [16]. Additional groups of ferrets challenged with low and high doses (i.e., 1 and 100 PFU) of EBOV via oronasal administration, while of interest, were excluded from this study as further investigation into this mucosal route was not a primary objective. Our results demonstrated that disease progression following oral and oronasal challenge was equivalent between sexes with no significant differences between route or dose. Most ferrets reached euthanasia criteria due to advanced clinical disease five to six days post-challenge. During peak disease, we consistently observed sustained fever, petechial rash, high viral loads, coagulation disorders (notably, prolonged coagulation times), clinical chemistry perturbations, hematology alterations including lymphocytopenia and thrombocytopenia, and pathological abnormalities. These results align with those previously reported from other ferret filovirus challenge studies involving mucosal (IN) and traditional (IM) routes of infection [7,9,12,15], thereby demonstrating the robustness of the model.

In the low dose (1 PFU) oral challenge group, one ferret presented with a delayed disease course. This ferret ultimately succumbed on Day 10 but demonstrated late onset fever and less severe symptoms including minimal clinical pathology changes. As animals were pair-housed, it cannot be ruled out that infection in this ferret was due to a transmission event originating from the cage mate, particularly since transmission is known to occur [11]. Given the social nature of ferrets, cohousing is preferred whenever possible. However, in order to further refine mucosal challenge models, future studies incorporating single housing are desirable.

All animals administered EBOV via the ocular route survived until scheduled study termination 28 days post-challenge and presented with minimal disease or lacked measures of disease altogether. Although not statistically significant, alterations in hematology parameters (specifically increased lymphocytes in the 10 and 100 PFU dose groups) and minor histopathological findings associated with inflammation suggest that a subclinical infection was established. One limitation to this hypothesis is that seroconversion post-challenge was not assessed. While of interest, we were unable to conduct seroconversion analyses primarily due to a lack of collected samples. In addition, at the time this study was conducted, available reagents to assess host response and the absence of an optimized assay hindered our ability to execute analyses. The absence of fulminant disease following ocular challenge may have been due to multiple factors. First, the challenge doses may have been too low to establish infection via this route. As indicated above, results in ferrets challenged with the highest EBOV dose (100 PFU) are suggestive of a subclinical infection. A higher challenge dose may have led to a more prominent disease such as that observed in ferrets administered virus via oronasal and oral routes. Second, the eye is an immune privileged site. While ferrets are susceptible to infection via this route with viruses such as influenza [20,21], ocular administration may be suboptimal for establishment of EBOV disease. Despite the lack of apparent disease following ocular challenge in our study, future studies investigating higher virus doses and/or compromised ocular mucosa may provide additional insight into the suitability of this route for further model development.

Taken together, our results support the utility of the ferret as a model for the study of Ebola virus disease following mucosal infection, particularly via oronasal and oral routes. As the need for medical countermeasures increases, these models may serve as critical tools for the advancement of new products.

## 4. Materials and Methods

### 4.1. Ethics Statement and Biocontainment

Study procedures were performed in accordance with relevant guidelines and regulations. Procedures involving animals complied with the Final Rules of the Animal Welfare Act regulations (9 CFR Parts 1, 2, and 3) and Guide for the Care and Use of Laboratory Animals: Eighth Edition (Institute of Laboratory Animal Resources, National Academies Press, 2011; the Guide). Certified LabDiet High Density Ferret Diet was provided to the animals daily. Drinking water (RO) was provided ad libitum through water bottles and an automatic watering system. To promote and enhance the psychological well-being of the animals, environmental enrichment consisting of ferret toys and habitat accessories (e.g., paper bags, tubes, etc.) was provided. This study was conducted in UTMB’s AAALAC (Association for the Assessment and Accreditation of Laboratory Animal Care)-accredited facility and was approved by UTMB’s Institutional Animal Care and Use Committee (IACUC). All biocontainment work was approved by UTMB’s Institutional Biosafety Committee. Biosafety Level 4 tasks were performed by appropriately trained personnel in the Galveston National Laboratory.

### 4.2. Ferrets

Twenty-eight ferrets (20–21 weeks old) weighing 0.78–0.94 kg (female) and 1.18–1.56 kg (male) were procured from Marshall BioResources (North Rose, NY, USA). Each animal was implanted subcutaneously by the vendor with a transponder chip (Bio Medic Data Systems [BMDS]) for identification and subcutaneous body temperature monitoring. Animals were randomized into seven groups (*n* = 4/group) by challenge dose and route such that each cage contained two ferrets of equal sex that were dosed with the same virus concentration and route (Table 4). To minimize bias, study personnel were blinded to group assignments.

For continuous core body temperature measurements, a DST micro-T implantable temperature logger (Star–Oddi, Gardabaer, Iceland) was surgically implanted into the peritoneal cavity of each animal; data recording was set to 15-min intervals.

### 4.3. Virus Challenge, Clinical Observations and Measurements, and Biosampling

#### 4.3.1. Virus Challenge

Ebola virus (EBOV, BEI Resources NR-50306) suspensions were prepared on the day of challenge. To maintain blinding, ferrets were administered virus or sterile Dulbecco’s phosphate buffered saline without divalent cations (DPBS) for each route specified in Table 1. Specifically, for each ferret, a dose kit was prepared that contained three pre-labeled tubes specifying the animal identification and dose route (i.e., oronasal, oral, and ocular). An individual unblinded to the groupings dispensed the EBOV suspension or DPBS into the appropriate tubes (*n* = 1 tube for the EBOV suspension and *n* = 2 tubes for the DPBS). Each tube was filled with an equivalent volume to maintain blinding of the dosing team. Following sedation via inhalational isoflurane, animals were administered doses via oronasal (0.25 mL per nostril and 0.5 mL to the epiglottis), oral (0.25 mL sublingual), and ocular (0.005 mL per conjunctiva) delivery; each ferret received material from each of the prepared tubes (*n* = 3 total). Material was delivered dropwise using a calibrated micropipette. For ocular delivery, material was gently massaged over the surface of the eye via the eyelid. Challenge doses were confirmed via plaque assay on Vero E6 cell monolayers as described previously [22].

#### 4.3.2. Clinical Observations and Scoring

Clinical observations were performed, at minimum, twice daily for up to 28 days post-challenge. Observations focused on appearance, activity and behavior, respiratory parameters, hydration status, neurological signs, and the presence of visible petechiae. Any animals exhibiting signs consistent with significant distress/moribundity and those that survived until study termination (Day 28) were humanely euthanized per the approved IACUC protocol.

#### 4.3.3. Body Weights and Subcutaneous Temperatures

Body weights and subcutaneous temperatures were measured daily through Day 10 and on Days 14, 21, and 28 (study termination). Subcutaneous body temperatures were measured at equivalent times each day via the implanted BMDS transponder chips. Additional terminal measurements were performed on animals that required euthanasia due to advanced clinical disease.

#### 4.3.4. Blood Collection and Clinical Pathology

On Days 0 (pre-challenge), 5, 10, 14, 21, and 28, blood was collected from the anterior vena cava into serum separator tubes and tubes containing anticoagulant (ethylenediaminetetraacetic acid [EDTA] and sodium citrate) for clinical chemistry, hematology, and coagulation analyses. Additional terminal collections were performed on animals that required euthanasia due to advanced clinical disease. Clinical chemistry was performed on harvested serum using Abaxis VetScan Comprehensive Diagnostic Profile reagent rotors (Abaxis, Inc., Union City, CA, USA) in conjunction with the Abaxis VetScan VS2^®^ Chemistry Analyzer (Abaxis, Inc.). Hematology was performed on EDTA blood using the Abaxis VETSCAN^®^ HM5 Hematology Analyzer (Abaxis, Inc.). Sodium citrate blood was analyzed for Activated Partial Thromboplastin Time (aPTT) and Prothrombin Time (PT) using the IDEXX Coag Dx™ Analyzer (IDEXX Laboratories, Inc., Westbrook, ME, USA) and associated aPTT/PT test cartridges. All clinical pathology instrumentation was operated per manufacturer instructions.

#### 4.3.5. Serum Viral Load

Serum viral load was assessed via plaque assay and qRT-PCR. Plaque assays were performed as described above for the virus challenge suspensions. For qRT-PCR analysis, 50 µL of serum was added to 250 µL of TRIzol LS (Life Technologies, Carlsbad, CA, USA) and stored at ≤ −65 °C until used for RNA extraction. RNA extraction and qRT-PCR were performed as described previously [23]. In brief, samples in TRIzol LS were processed to RNA using Zymo Direct-zol™ RNA Mini Prep (Zymo Research, Irvine, CA, USA) kits per manufacturer instructions. RNA samples were analyzed via qRT-PCR targeting the EBOV glycoprotein gene (GeneBank accession AF086833). Primers were obtained from Invitrogen and probe was obtained from Integrated DNA Technologies. Probe was labeled as the 5′-end with fluorophore 9-carboxyfluroescein (6-FAM) and included a 3′-end nonfluorescent quencher/Minor Groove Binder (MGB). Master Mix was prepared by combining forward primer (1000 nM, 5′-TTTTCAATCCTCAACCGTAAGGC-3′), reverse primer (1000 nM, 5′-CAGTCCGGTCCCAGAATGTG-3′), and probe (100 nM, 5′-6FAM-CATGTGCCGCCCCATCGCTGC-MGBNFQ-3′) with 12.5 µL of 2X QuantiFast Probe Mix (QIAGEN, Hilden, Germany), 0.25 µL of 2X QuantiFast RT Mix (QIAGEN), 1.5 µL of 50 mM MgSO_4_ and PCR-grade water (fill to 20 µL) for each planned reaction. To the Master Mix, test sample (5 µL) was added resulting in a final volume of 25 µL per reaction. Real-time analysis was performed using the Bio-Rad CFX96™ Real-Time PCR Detection System (Bio-Rad Laboratories, Hercules, CA, USA). Thermocycling conditions were as follows: Step 1, 1 cycle, 50 °C for 15 min; Step 2, 1 cycle, 95 °C for 5 min; Steps 3–5, 45 cycles, 95 °C for 10 s, 60 °C for 30 s, single read. For quantification purposes, a HPLC-purified synthetic EBOV RNA standard containing the conserved EBOV glycoprotein sequence was used (5′-CAGUCCGGUCCCAGAAUGUGGCAUGUGCCGCCCCAUCGCUGCAGCAAGAAAUCAAUUGCCUUACGGUUGAGGAUUGAAAA-3′).

### 4.4. Necropsy and Tissue Processing

A gross necropsy was conducted on all animals following scheduled and unscheduled euthanasia. Gross necropsies included examination of the external surface of the body, all external orifices, the thoracic and abdominal cavities and their contents. Representative samples of liver, kidney (right), lung, spleen, brain, mesenteric lymph node, rectum, colon, tongue (sublingual tissue), and eye were collected for viral load and histopathology. In addition to tissues, a nasal swab sample was collected from each ferret and placed into 1 mL of sterile phosphate buffered saline. For viral load analysis, tissue samples were disrupted using a QIAGEN TissueLyser II per manufacturer instructions. Following disruption, samples were clarified via centrifugation (10,000× *g* for 10 min). Clarified tissue supernatant and nasal swab wash were processed for plaque assay and qRT-PCR as already described. For histopathology, tissues were placed in 10% neutral buffered formalin and were allowed to fix (with changeout) for a minimum of 30 days prior to removal from the BSL-4 laboratory. Fixed tissues were processed to hematoxylin and eosin-stained slides and examined by a board-certified pathologist at Experimental Pathology Laboratories, Inc. (EPL^®^, Steriling, VA, USA). The pathologist was blinded to groups during microscopic evaluation. A key identifying animal groupings was provided after completion of the microscopic evaluation.

### 4.5. Statistical Analyses

Statistical analyses were performed to characterize and compare time to death between the three challenge routes and evaluate dose–response for oral and ocular routes. Kaplan–Meier plots were prepared and median time to death was estimated with 95% confidence intervals (if calculable given small sample sizes). Cox proportional hazards regression was used to compare challenge routes and evaluate dose–response.

Statistical analysis was performed at scheduled time points and terminal measurements for animals that were euthanized. For each endpoint, ANOVA models were fitted separately to the change from baseline data at each time point to determine if there were significant changes from the pre-challenge baseline within a group, or differences among the groups. Tukey’s multiple comparisons were used to test for significant differences between pairs of groups. The analysis was performed at each scheduled collection time and for the combined terminal measurements, if at least 3 groups with at least 2 animals per group were available for analysis. Two-way ANOVA models were also fitted separately to the change from baseline data at each time point to determine the effects of log-10 transformed challenge dose and challenge route on each endpoint when possible.

Median time to onset of elevated temperature was calculated for each group based on core body temperature readings collected every 15 min from implanted temperature loggers, where elevated temperature was defined as 2 consecutive hours (8 consecutive measurements) of temperatures greater than 40 °C. Ninety-five percent (95%) confidence intervals were also calculated.

## Figures and Tables

**Table 1:** Clinical signs following EBOV mucosal challenge in ferrets ^1^.

Route	Dose ^2^	Lethargy/Inactivity	Unkempt Appearance	Respiration >50 BPM ^3^	Diarrhea/Loose Stool
Oronasal	10 PFU	4/4	2/4	1/4	0/4
Oral	1 PFU	4/4	4/4	4/4	0/4
Oral	10 PFU	4/4	2/4	1/4	2/4
Oral	100 PFU	4/4	3/4	3/4	0/4
Ocular	1 PFU	0/4	0/4	0/4	0/4
Ocular	10 PFU	0/4	0/4	0/4	0/4
Ocular	100 PFU	0/4	0/4	0/4	0/4

^1^ Results are presented as the number of animals in which the specified clinical sign was noted out of the total number of animals in the challenge route/dose group. ^2^ PFU, plaque forming units. ^3^ BPM, breaths per minute.

**Table 2:** Predominant macroscopic (gross) findings following EBOV mucosal challenge in ferrets ^1^.

Route	Diffuse, Pinpoint, Red Discoloration						Pale/Yellow Liver	Enlarged/Dark Lymph Nodes	Mottled Dark Red Lungs	Mottled Dark Red Spleen
	Skin	Bladder Mucosa	Stomach Mucosa	Spleen	Rectum	Thymus				
Oronasal	4/4	2/4	1/4	0/4	3/4	2/4	4/4	2/4	2/4	0/4
Oral	12/12	8/12	6/12	4/12	8/12	2/12	12/12	6/12	2/12	2/12
Ocular	0/12	0/12	0/12	0/12	0/12	0/12	0/12	0/12	2/12	2/12

^1^ Results are presented as the number of animals in which the specified gross finding was noted out of the total number of animals in the challenge route group.

**Table 3:** Predominant histopathological findings following EBOV mucosal challenge in ferrets ^1^.

Organ	Finding	Oronasal	Oral			Ocular		
		10 PFU ^2^	1 PFU ^2^	10 PFU ^2^	100 PFU ^2^	1 PFU ^2^	10 PFU ^2^	100 PFU ^2^
Liver	Hepatocellular degeneration	4/4	4/4	4/4	3/4	0/4	1/4	0/4
	Inflammation	4/4	4/4	3/4	3/4	0/4	1/4	0/4
	Hepatocellular necrosis	3/4	3/4	4/4	3/4	0/4	0/4	0/4
	Vacuolation	3/4	2/4	4/4	2/4	0/4	0/4	0/4
Lung	Perivascular edema	4/4	3/4	3/4	3/4	0/4	1/4	0/4
	Alveolar inflammation	3/4	3/4	1/4	3/4	0/4	3/4	2/4
	Perivascular inflammation	4/4	3/4	3/4	3/4	0/4	1/4	0/4
	Perivascular necrosis	1/4	0/4	0/4	1/4	0/4	0/4	0/4
Spleen	Decreased lymphocytes	4/4	0/4	1/4	1/4	0/4	0/4	0/4
	Inflammation	4/4	4/4	4/4	3/4	0/4	1/4	0/4
	Necrosis	4/4	3/4	3/4	2/4	0/4	0/4	0/4
Kidney	Tubule vacuolation	3/4	3/4	4/4	1/4	0/4	1/4	0/4
Bladder	Mucosal hemorrhage	1/4	0/4	0/4	0/4	0/4	0/4	0/4
Stomach	Mucosal hemorrhage	1/4	0/4	0/4	0/4	0/4	0/4	0/4
Lower GI ^3^	Mucosal hemorrhage	0/4	0/4	1/4	0/4	0/4	0/4	0/4
Brain	Hippocampus necrosis	1/4	0/4	1/4	0/4	0/4	0/4	0/4

^1^ Results are presented as the number of animals in which the specified histopathological finding was noted out of the total number of animals in the challenge dose/route group. ^2^ Target challenge dose; PFU, plaque forming units. ^3^ GI, gastrointestinal tract; includes rectum and/or colon.

**Table 4:** Randomization and Group Allocation.

Group	Number ^1^	Route	Challenge ^2^
1	2M/2F	Oronasal	10 PFU
2	2M/2F	Oral	1 PFU
3	2M/2F	Oral	10 PFU
4	2M/2F	Oral	100 PFU
5	2M/2F	Ocular	1 PFU
6	2M/2F	Ocular	10 PFU
7	2M/2F	Ocular	100 PFU

^1^ M, male; F, female. ^2^ PFU, plaque forming units.

## Data Availability

The data presented in this study are available in the article.

## Supplementary Materials

The following are available online at https://www.mdpi.com/2076-0817/10/3/292/s1, Figure S1: Total viral loads in tissue.

## References

[1] Bennett R.S., Huzella L., Jahrling P.B., Bollinger L., Olinger G.G., Hensley L.E. (2017). Nonhuman Primate Models of Ebola Virus Disease. Curr. Top. Microbiol. Immunol..

[2] Geisbert T.W., Pushko P., Anderson K., Smith J., Davis K.J., Jahrling P.B. (2002). Evaluation in Nonhuman Primates of Vaccines against Ebola Virus. Emerg. Infect. Dis..

[3] Jaax N.K., Davis K.J., Geisbert T.J., Vogel P., Jaax G.P., Topper M., Jahrling P.B. (1996). Lethal experimental infection of rhesus monkeys with Ebola-Zaire (Mayinga) virus by the oral and conjunctival route of exposure. Arch. Pathol. Lab. Med..

[4] St Claire M.C., Ragland D.R., Bollinger L., Jahrling P.B. (2017). Animal Models of Ebolavirus Infection. Comp. Med..

[5] Bente D., Gren J., Strong J.E., Feldmann H. (2009). Disease modeling for Ebola and Marburg viruses. Dis. Model. Mech..

[6] Envigo (2020). Non-Human Primate Shortage Is Forecasted to Accelerate. Planning Ahead Is Key to Circumvent Potential Disruptions in Research.

[7] Cross R.W., Fenton K.A., Geisbert T.W. (2018). Small animal models of filovirus disease: Recent advances and future directions. Expert Opin. Drug Discov..

[8] Siragam V., Wong G., Qiu X.G. (2018). Animal models for filovirus infections. Zool Res..

[9] Cross R.W., Mire C.E., Borisevich V., Geisbert J.B., Fenton K.A., Geisbert T.W. (2016). The Domestic Ferret (*Mustela putorius* furo) as a Lethal Infection Model for 3 Species of Ebola virus. J. Infect. Dis..

[10] Cross R.W., Speranza E., Borisevich V., Widen S.G., Wood T.G., Shim R.S., Adams R.D., Gerhardt D.M., Bennett R.S., Honko A.N. (2018). Comparative Transcriptomics in Ebola Makona-Infected Ferrets, Nonhuman Primates, and Humans. J. Infect. Dis..

[11] De La Vega M.A., Soule G., Tran K.N., Tierney K., He S., Wong G. (2018). Modeling Ebola Virus Transmission Using Ferrets. mSphere.

[12] Kozak R., He S., Kroeker A., De La Vega M.-A., Audet J., Wong G., Urfano C., Antonation K., Embury-Hyatt C., Kobinger G.P. (2016). Ferrets Infected with Bundibugyo Virus or Ebola Virus Recapitulate Important Aspects of Human Filovirus Disease. J. Virol..

[13] Wong G., Leung A., He S., Cao W., De La Vega M.-A., Griffin B.D., Soule G., Kobinger G.P., Kobasa D., Qiu X. (2018). The Makona Variant of Ebola Virus Is Highly Lethal to Immunocompromised Mice and Immunocompetent Ferrets. J. Infect. Dis..

[14] Bornholdt Z.A., Herbert A.S., Mire C.E., He S., Cross R.W., Wec A.Z., Abelson D.M., Geisbert J.B., James R.M., Rahim N. (2019). A Two-Antibody Pan-Ebolavirus Cocktail Confers Broad Therapeutic Protection in Ferrets and Nonhuman Primates. Cell Host Microbe.

[15] Kroeker A., He S., De La Vega M.-A., Wong G., Embury-Hyatt C., Qiu X. (2017). Characterization of Sudan Ebolavirus infection in ferrets. Oncotarget.

[16] Marsh G. The potential of the ferret model. Proceedings of the Filovirus Animal Nonclinical Group (FANG)—World Health Organization (WHO) Medical Countermeasures (MCM) Workshop.

[17] Beeching N.J., Fenech M., Houlihan C.F. (2014). Ebola virus disease. BMJ.

[18] Fletcher T.E., Fowler R.A., Beeching N.J. (2014). Understanding organ dysfunction in Ebola virus disease. Intensiv. Care Med..

[19] Yan F., He S., Banadyga L., Zhu W., Zhang H., Rahim N., Collignon B., Senthilkumaran C., Embury-Hyatt C., Qiu X. (2019). Characterization of Reston virus infection in ferrets. Antivir. Res..

[20] Belser J.A., Gustin K.M., Maines T.R., Pantin-Jackwood M.J., Katz J.M., Tumpey T.M. (2012). Influenza Virus Respiratory Infection and Transmission Following Ocular Inoculation in Ferrets. PLoS Pathog..

[21] Belser J.A., Gustin K.M., Katz J.M., Maines T.R., Tumpey T.M. (2014). Influenza Virus Infectivity and Virulence following Ocular-Only Aerosol Inoculation of Ferrets. J. Virol..

[22] Shurtleff A., Biggins J., Keeney A., Zumbrun E., Bloomfield H., Kuehne A., Audet J., Alfson K., Griffiths A., Olinger G. (2012). Standardization of the Filovirus Plaque Assay for Use in Preclinical Studies. Viruses.

[23] Comer J.E., Escaffre O., Neef N., Brasel T., Juelich T.L., Smith J.K., Smith J., Kalveram B., Perez D.D., Massey S. (2019). Filovirus Virulence in Interferon α/β and γ Double Knockout Mice, and Treatment with Favipiravir. Viruses.

