# Supplementary material for: Mucosal Challenge Ferret Models of Ebola Virus Disease
Source: Pathogens. 2021 Mar 4;10(3):292. doi: 10.3390/pathogens10030292 (PMC8001755; doi:10.3390/pathogens10030292)
Supplement: Supplementary file 1 [file pathogens-10-00292-s001.pdf]

pathogens

MDPI

Article

Mucosal Challenge Ferret Models of Ebola Virus Disease

Trevor Brasel [1*], Jason E. Comer [1], Shane Massey [1], Jeanon Smith [1], Jennifer Smith [2], Matthew Hyde [3], Andrew Kocsis [3], Melicia Gainey [4], Nancy Niemuth [4], Cheryl Triplett[4] and Thomas Rudge, Jr. [4]

1 University of Texas Medical Branch, Department of Microbiology and Immunology, 301 University Blvd., Galveston, TX, 77573; trbrasel@utmb.edu; jscomer@utmb.edu; chmassey@utmb.edu; jensmit1@utmb.edu
2 University of Texas Medical Branch, Department of Pathology, 301 University Blvd., Galveston, TX, 77573; jeksmith@utmb.edu
3 University of Texas Medical Branch, Animal Resources Center, 301 University Blvd., Galveston, TX, 77573; mahyde@utmb.edu; agkocsis@utmb.edu
4 Battelle; 1425 Plain City-Georgesville Road, NE, West Jefferson, OH, 43162; GaineyM@battelle.org; niemuth@battelle.org; triplettc@battelle.org; rudget@battelle.org
* Correspondence: trbrasel@utmb.edu; Tel.: +1-409-266-6907

Supplemental Material

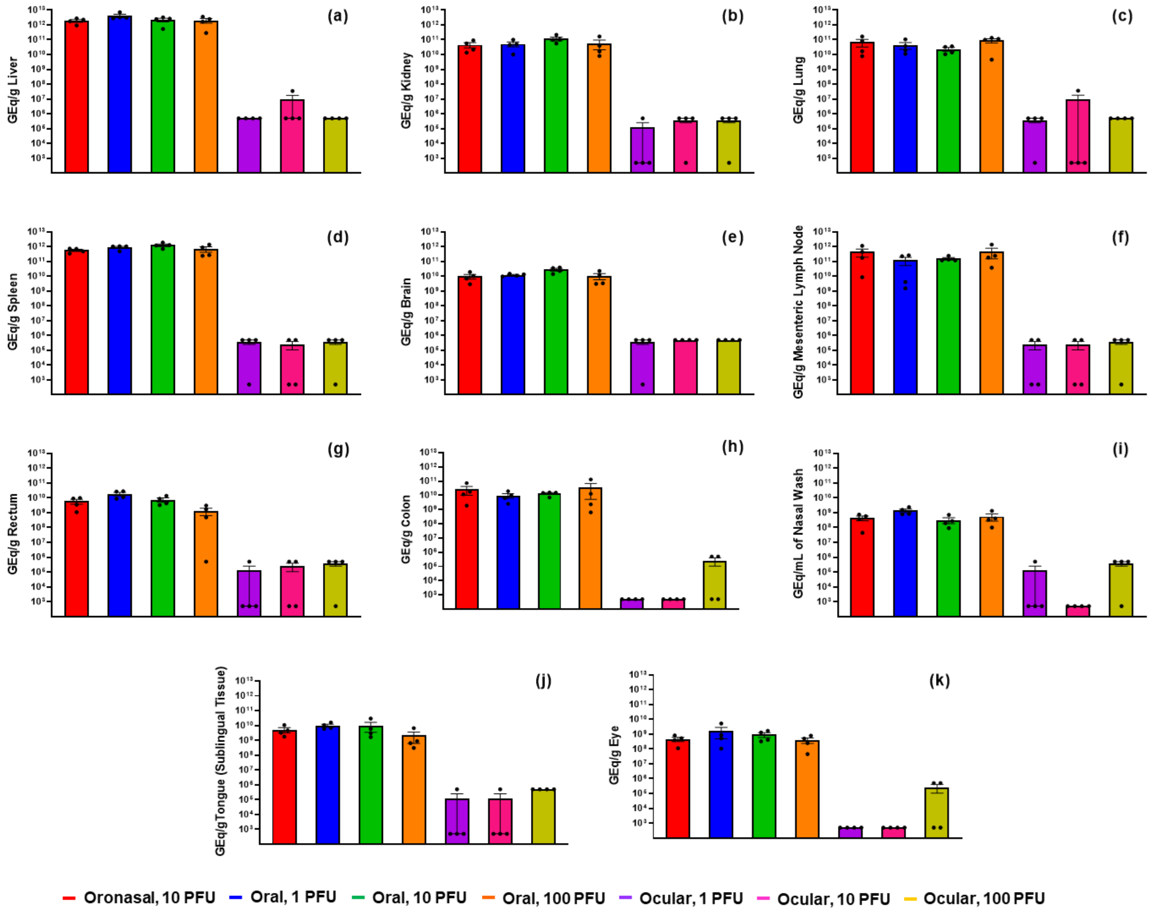

Figure 1. Viral RNA in tissue. From each ferret, select tissues and nasal wash were collected and analyzed for EBOV via qRT-PCR. (**a**) Liver; (**b**) kidney; (**c**) lung; (**d**) spleen; (**e**) brain; (**f**) mesenteric lymph node; (**g**) rectum; (**h**) colon; (**i**) nasal wash; (**j**) tongue [sublingual tissue]; (**k**) eye. Error bars represent standard error of the mean. GEq/g, EBOV genome equivalents per gram of tissue. Samples

that lacked detectable signal following PCR amplification were assigned a value of 500 GEq/g (or mL for nasal wash), representing one-half the limit of detection on a per mL basis. Amplified samples that quantified to values below the lowest tested standard (1,000 GEq/μL) were assigned a value of 5 x 10^5 GEq/g (or mL for nasal wash), representing one-half the lower limit of quantitation on a per mL basis.
